# Knee pain as a predictor of structural progression over 4 years: data from the Osteoarthritis Initiative, a prospective cohort study

**DOI:** 10.1186/s13075-018-1751-4

**Published:** 2018-11-06

**Authors:** Yuanyuan Wang, Andrew J. Teichtahl, François Abram, Sultana Monira Hussain, Jean-Pierre Pelletier, Flavia M. Cicuttini, Johanne Martel-Pelletier

**Affiliations:** 1 Department of Epidemiology and Preventive Medicine, School of Public Health and Preventive Medicine, Monash University, 553 St Kilda Road, Melbourne, VIC 3004 Australia; 2 Medical Imaging Research & Development, ArthroLab Inc., Montreal, QC Canada; 3 Osteoarthritis Research Unit, University of Montreal Hospital Research Centre (CRCHUM), Montreal, QC Canada

**Keywords:** Pain, Knee osteoarthritis, Cartilage, Incidence, Progression, Magnetic resonance imaging

## Abstract

**Background:**

There is evidence that knee pain not only is a consequence of structural deterioration in osteoarthritis (OA) but also contributes to structural progression. Clarifying this is important because targeting the factors related to knee pain may offer a clinical approach for slowing the progression of knee OA. The aim of this study was to examine whether knee pain over 1 year predicted cartilage volume loss, incidence and progression of radiographic osteoarthritis (ROA) over 4 years.

**Methods:**

Osteoarthritis Initiative participants with no ROA (Kellgren-Lawrence grade ≤ 1) (*n* = 2120) and with ROA (Kellgren-Lawrence grade > 2) (*n* = 2249) were examined. Knee pain was assessed at baseline and 1 year using the Western Ontario and McMaster Universities Osteoarthritis Index (WOMAC). Knee pain patterns were categorised as no pain (WOMAC pain < 5 at baseline and 1 year), fluctuating pain (WOMAC pain > 5 at either time point) and persistent pain (WOMAC pain > 5 at both time points). Cartilage volume, incidence and progression of ROA were assessed using magnetic resonance imaging and x-rays at baseline and 4-years.

**Results:**

In both non-ROA and ROA, greater baseline WOMAC knee pain score was associated with increased medial and lateral cartilage volume loss (*p* ≤ 0.001), incidence (OR 1.07, 95% CI 1.01–1.13) and progression (OR 1.07, 95% CI 1.03–1.10) of ROA. Non-ROA and ROA participants with fluctuating and persistent knee pain had increased cartilage volume loss compared with those with no pain (*p* for trend ≤ 0.01). Non-ROA participants with fluctuating knee pain had increased risk of incident ROA (OR 1.62, 95% CI 1.04–2.54), corresponding to a number needed to harm of 19.5. In ROA the risk of progressive ROA increased in participants with persistent knee pain (OR 1.82, 95% CI 1.28–2.60), corresponding to a number needed to harm of 9.6.

**Conclusions:**

Knee pain over 1 year predicted accelerated cartilage volume loss and increased risk of incident and progressive ROA. Early management of knee pain and controlling knee pain over time by targeting the underlying mechanisms may be important for preserving knee structure and reducing the burden of knee OA.

**Electronic supplementary material:**

The online version of this article (10.1186/s13075-018-1751-4) contains supplementary material, which is available to authorized users.

## Background

Pain and structural articular degeneration are major clinical manifestations of knee osteoarthritis (OA). Although previous studies have predominantly focussed on whether structural disease progression predicts knee pain in people with knee OA [1–4], there have been relatively few studies examining whether knee pain is a predictor of structural progression of knee OA [5, 6]. There is increasing evidence for an important interplay between joint structures such as cartilage, bone, muscle and other soft tissues in maintaining joint health [7]. Pain through mechanisms such as inflammation and reduced mobility can adversely affect these joint structures, resulting in structural progression [8, 9]. Thus it is plausible that knee pain not only is a consequence of structural deterioration in OA but also contributes to structural progression. Clarifying this is important, because if this is the case, targeting the factors related to knee pain may offer a potential strategy for slowing disease progression of OA.

The major structural outcomes commonly examined in the development and progression of knee OA include cartilage volume loss assessed by magnetic resonance imaging (MRI) and incidence and progression of radiographic osteoarthritis (ROA). The findings of prospective cohort studies examining whether knee pain is a predictor of structural progression are summarised in Table 1. Inconsistent results have emerged regarding whether knee pain predicts cartilage volume/thickness loss [1, 5, 10–14]. This may be attributable to small to moderate sample sizes, different study populations, subgroup analyses and varied outcome measures. Although some studies found no association between baseline knee pain and subsequent cartilage volume loss in symptomatic knee OA [1, 10, 13] or asymptomatic [11] individuals, other studies showed relationships of baseline knee pain [14], frequent knee pain [5] and change in knee pain [1, 12, 13] with cartilage volume/thickness loss. In terms of studies with radiographic outcomes, some studies have suggested no association between baseline knee pain and progression of ROA [6, 15–17], whereas other studies have reported associations of baseline knee pain with incident ROA [16, 18], incident accelerated knee OA [19] and progressive ROA [18]. Differences in study population, assessment of knee pain, duration of follow-up and definition of incidence and progression of knee ROA may provide potential explanations of the inconclusive results. Larger cohort studies with longer follow-up have shown significant associations between knee pain and incidence and/or progression of knee ROA [16, 18, 19].

**Table 1:** Prospective cohort studies examining knee pain as a predictor of knee structural outcomes

Author, year	Participants	Exposure	Outcome measure	Main results
Outcome: change in cartilage volume or thickness				
Raynauld et al., 2004 [10]	40 patients with symptomatic knee OA 2-year follow-up (*N* = 32)	WOMAC pain	Cartilage volume loss; slow (< 2% global cartilage loss) and rapid (> 15% global cartilage loss) progressors	Trend for higher baseline knee pain in rapid progressors than in slow progressors (49.9 ± 6.1 vs. 34.0 ± 4.4, *p* = 0.05)
Wluka et al., 2004 [11]	81 healthy post-menopausal women 2.5-year follow-up (*N* = 57)	WOMAC pain	Change in tibial cartilage volume	Baseline knee pain not associated with change in tibial cartilage volume (data not shown)
Wluka et al., 2004 [1]	132 people with symptomatic knee OA 2-year follow-up (*N* = 117)	WOMAC pain	Change in tibial cartilage volume	The severity of baseline knee pain did not predict subsequent tibial cartilage volume loss (*r* = 0.13, *p* = 0.14) There was a weak association between worsening of knee pain and increased tibial cartilage volume loss (*r* = 0.28, *p* = 0.002)
Raynauld et al., 2006 [12]	110 patients with symptomatic knee OA 2-year follow-up (*N* = 107)	WOMAC pain	Change in cartilage volume for the entire knee (global) and medial and lateral knee compartments	Medial compartment cartilage volume loss was associated with simultaneous knee pain change at 2 years (β coefficient − 0.45, *p* = 0.03)
Pelletier et al., 2007 [13]	110 patients with symptomatic knee OA 2-year follow-up (*N* = 107)	WOMAC pain	Change in knee cartilage volume from subregions	Baseline WOMAC pain not associated cartilage volume loss in medial central femoral condyle or medial central tibial plateau An increase in WOMAC pain score associated with cartilage volume loss in medial central tibial plateau (β coefficient − 0.26, *p* = 0.007)
Eckstein et al., 2011 [5]	718 participants with radiographic knee OA (K-L grades 2–4) 12-month follow-up	Questionnaire categorized as no, infrequent or frequent pain	Change in cartilage thickness in the central subregion of medial weight-bearing femoral condyle	Change in cartilage thickness − 12 μm in knees without pain vs − 54 μm in those with frequent pain at baseline, *p* = 0.01 The percentage of “progressors” (knees with cartilage thinning) was greater in knees with frequent pain than in those without pain for total joint (29% vs 16%, *p* = 0.004) and medial compartment (23% vs 13%, *p* = 0.015), but not for lateral compartment (20% vs 16%, *p* = 0.29)
Saunders et al., 2012 [14]	912 randomly selected individuals, 53% had radiographic OA in the medial compartment, 24% had radiographic OA in the lateral compartment 2.9-year follow-up (*N* = 399)	WOMAC pain	Change in tibial cartilage volume	Pain independently predicted lateral tibial cartilage volume loss. WOMAC knee pain: B − 0.14 (95% CI, − 0.22, − 0.05); knee pain yes/no: B − 0.96 (95% CI, − 1.91, − 0.00). No significant associations for medial tibial cartilage volume loss
Outcome: radiographic incidence or progression of OA				
Spector et al., 1992 [15]	169 patients with OA of the hands or knees 11-year follow-up (*N* = 63)	VAS pain	Radiographic progression defined by > 10% reduction in joint space width or a K-L grade increase ≥ 1	9 of 15 people with knee pain at baseline had radiographic progression, compared with 6 of 16 without knee pain at baseline (*p* = 0.20)
Cooper et al., 2000 [16]	583 people from a population cohort 5.1-year follow-up (*N* = 354)	Have you had pain in or around your knee on most days for at least 1 month, at some time during the last year?	Incident and progressive OA defined using thresholds of both K-L grade 2 and K-L grade 1	Knee pain associated with incident OA when defined by K-L ≥ 1 (OR 2.9, 95% CI 1.2–6.7) but not K-L ≥ 2 (OR 1.3, 95% CI 0.6–2.7) No association between knee pain and progressive OA defined by either K-L ≥ 1 (OR 0.8, 95% CI 0.4–1.7) or K-L ≥ 2 (OR 2.4, 95% CI 0.7–8.0)
Wolfe et al., 2002 [17]	1507 patients with symptomatic knee OA	VAS pain	Joint space narrowing score = 3	Knee pain not associated with progression to maximum joint space narrowing (data not shown)
Miyazaki et al., 2002 [6]	106 patients with medial compartment knee OA 6-year follow-up (*N* = 74)	Knee rating system of the Hospital for Special Surgery	Radiographic progression defined as > 1 grade increase in narrowing of joint space width of the medial compartment	Baseline knee pain not associated with radiographic progression (OR 0.93, 95% CI 0.78–1.11, *p* = 0.43)
Mazzuca et al., 2005 [46]	174 obese women with unilateral knee OA 16- and 30-month follow-up	WOMAC pain	Joint space narrowing > 0.50 mm	Baseline WOMAC pain > 11 as a predictor of joint space narrowing > 0.50 mm: Month 16 (*n* = 73): sensitivity 77%, positive predictive value 36%, specificity 59%, negative predictive value 89% Month 30 (*n* = 70): sensitivity 65%, positive predictive value 45%, specificity 62%, negative predictive value 78%
Muraki et al., 2012 [18]	3040 people from a population-based cohort 3.3-year follow-up (*N* = 2262)	Have you experienced right/left knee pain on most days in the past month, in addition to now?	Incident radiographic knee OA; progressive radiographic knee OA	Knee pain at baseline associated with incident K-L grade > 3 knee OA (OR 2.53, 95% CI 1.59–4.00) and progressive radiographic knee OA (OR 2.63, 95% CI 1.81–3.81), but not incident K-L grade > 2 knee OA
Driban et al., 2016 [19]	1930 participants with no radiographic knee OA in either knee 4-year follow-up	WOMAC pain	Incident accelerated knee OA: at least one knee progressed to end-stage knee OA (K-L grade > 3)	Individuals with accelerated knee OA had greater WOMAC pain (OR 2.00, 95% CI 1.33–3.00)

*Abbreviations: K-L* Kellgren Lawrence, *OA* Osteoarthritis, *VAS* Visual analogue scale, *WOMAC* Western Ontario and McMaster Universities Osteoarthritis Index

The National Institutes of Health Osteoarthritis Initiative (OAI) is the largest observational cohort of knee OA [20] and offers the opportunity to examine whether knee pain predicts structural progression. The aim of the present study was to examine whether baseline knee pain and knee pain patterns over 1 year are predictors of cartilage volume loss, incidence and progression of ROA over 4 years in a large cohort of individuals with and without knee ROA.

## Methods

### Osteoarthritis Initiative

Data were extracted from the OAI database, which holds data derived from a publicly available, multicentre, population-based cohort study of knee OA (https://oai.nih.gov). The OAI comprises data of 4796 participants aged 45–79 years with or at risk for knee OA at baseline. OAI exclusion criteria were inflammatory arthritis, severe joint space narrowing in both knees, unilateral knee replacement and severe joint space narrowing in the contralateral knee, inability to undergo MRI or provide a blood sample, use of walking aids except a single straight cane ≤ 50% of the time, or unwillingness to provide informed consent. Participants were recruited at four clinical sites, and the study was approved by the institutional review board at each of the sites. All participants gave informed consent.

### Participants of the current study

Bilateral standing posteroanterior fixed-flexion knee radiographs [21] were assessed for baseline Kellgren-Lawrence (K-L) grading (0–4) (*n* = 4369). If both knees had no evidence of ROA, the dominant knee was selected for analyses. If only one knee had evidence of ROA, this was the selected knee for analyses. If both knees had evidence of ROA, the most severe knee was selected for analyses. When the severity was equal between sides, the most painful knee was selected for analyses. In the case of equal pain in both knees, the dominant knee was selected for analyses. Participants had been categorized into two groups based on their baseline K-L grade as part of their participation in the study: non-ROA (incidence cohort) defined by a baseline K-L grade ≤ 1 (*n* = 2120) and ROA (progression cohort) defined by a baseline K-L grade ≥ 2 (*n* = 2249).

### Knee pain assessment

Knee pain was assessed yearly using the Western Ontario and McMaster Universities Osteoarthritis Index (WOMAC) pain subscale [22], Likert scale version. It consists of five items with scores ranging 0 to 20 and 20 being the worst pain. “Symptomatic” was defined as a WOMAC pain score > 5 based on the Low-Intensity Symptom State-Attainment Index cut-off [23]. This definition has been used in a previous OAI study in which WOMAC knee pain score > 5 represented the upper tertile of all participants with any pain in the cohort [24]. The knee pain patterns from baseline to 1-year follow-up were categorised as follows: no knee pain (WOMAC pain < 5 at both baseline and 1 year), fluctuating knee pain (WOMAC pain > 5 at either baseline or 1 year) and persistent knee pain (WOMAC pain > 5 at both baseline and 1 year).

### Cartilage volume assessment

Knee MRI was performed for the target knee using a 3-T apparatus (Magnetom Trio; Siemens, Erlangen, Germany). Cartilage volume was measured by sagittal double-echo steady-state imaging for medial and lateral tibiofemoral compartments (condyle and plateau) using an automatic human cartilage segmentation (ArthroLab, Montreal, QC, Canada) as previously described and validated [25, 26]. The test-retest revealed an excellent measurement error of 0.3 ± 1.6%, corresponding to a measurement error of 30.3 ± 126.2 mm^3^ [26]. The annual rate of cartilage volume loss over 4 years was obtained by calculating (4-year follow-up volume − baseline volume)/baseline volume/4, expressed as a percentage.

### Assessment of incidence and progression of ROA

Incidence of ROA was defined by a baseline K-L grade of 0 or 1 and a K-L grade ≥ 2 at 4-year follow-up. Progression of ROA was defined by a baseline K-L grade of 2 or 3 and an increase in K-L grade ≥ 1 at 4-year follow-up.

### Statistical analyses

Demographic, clinical, radiological and MRI data were systematically entered into a computerized database. Participant characteristics were compared between participants with and without ROA using independent samples *t* tests or chi-square tests when appropriate. With 2120 non-ROA participants, our study had 80% power to detect a regression coefficient as low as 0.006 between baseline knee pain and cartilage volume loss with five predictors, and a relative risk as low as 1.41 between baseline knee pain and incidence of ROA, α error of 0.05, two-sided significance. With 2249 participants with ROA, our study had 80% power to detect a regression coefficient as low as 0.0057 between baseline knee pain and cartilage volume loss with five predictors, and a relative risk as low as 1.27 between baseline knee pain and progression of ROA, α error of 0.05, two-sided significance. The association between baseline knee pain and cartilage volume loss was examined using multiple linear regression. The association between knee pain patterns over 1 year and cartilage volume loss was examined using the *F*-test (generalised linear model) with estimated marginal means (SE), and linear trend was assessed using multiple linear regression. The associations of baseline knee pain and knee pain patterns over 1 year with incidence and progression of ROA were examined using binary logistic regression. The attributable risk and number needed to harm (NNH) were calculated. NNH is a measure of how many people need to be exposed to a risk factor in order for one person to have a particular adverse effect. All the analyses were adjusted for gender, baseline age, body mass index (BMI) and K-L grade. All tests were two-sided, and *p* < 0.05 was considered statistically significant. Statistical analyses were performed using the IBM SPSS Statistics software package (version 24; IBM, Armonk, NY, USA).

## Results

Participant characteristics at baseline, as well as knee pain and structure changes over time, are shown in Table 2. Compared with non-ROA participants, participants with ROA were older, had higher BMIs and WOMAC pain scores, were more likely to have fluctuating and persistent knee pain, and had a greater rate of cartilage volume loss (all *p* < 0.001). The incidence and progression of ROA were 9.6% and 17.8%, respectively. There was no significant difference in age, gender or BMI between those who completed (*n* = 3395) and those who did not complete (*n* = 974) the 4-year follow-up. The non-completers had higher K-L grade and worse knee pain and were more likely to have ROA than the completers (all *p* < 0.05).

**Table 2:** Characteristics of study participants

	Non-ROA *n* = 2120	ROA *n* = 2249	*p* Value
Baseline characteristics			
Age, years	59.9 (9.1)	62.7 (8.9)	< 0.001
Female, *n* (%)	1224 (57.7)	1311 (58.3)	0.71
Body mass index, kg/m^2^	27.6 (4.5)	29.8 (4.8)	< 0.001
Kellgren-Lawrence grade, *n* (%)			–
0	1432 (67.5)	–	
1	688 (32.5)	–	
2	–	1173 (52.2)	
3	–	787 (35.0)	
4	–	289 (12.8)	
WOMAC pain score (range 0–20)	1.9 (2.7)	3.7 (3.8)	< 0.001
WOMAC pain score > 5, *n* (%)	307 (14.5)	760 (33.8)	< 0.001
Change in knee pain and structure			
Knee pain pattern over 1 year, *n* (%)			< 0.001
No knee pain at both baseline and 1 year	1669 (80.5)	1265 (57.8)	
Fluctuating knee pain (pain at either baseline or 1 year)	274 (13.2)	464 (21.2)	
Persistent knee pain (pain at both baseline and 1 year)	131 (6.3)	459 (21.0)	
Annual percentage cartilage volume loss over 4 years			
Medial compartment	0.68 (1.13)	1.36 (1.95)	< 0.001
Lateral compartment	0.73 (1.03)	1.21 (1.41)	< 0.001
Incidence of ROA over 4 years, *n* (%)	165 (9.6)	–	–
Progression of ROA over 4 years, *n* (%)	–	272 (17.8)	–

*ROA* Radiographic osteoarthritis, *WOMAC* Western Ontario and McMaster Universities Osteoarthritis Index

Data displayed as mean (SD) or number (%)

### Associations between baseline knee pain and structural progression over 4 years

In non-ROA, a greater baseline WOMAC pain score was associated with increased rate of cartilage volume loss in medial (regression coefficient 0.04%, 95% CI 0.02–0.06%) and lateral (0.04%, 0.02–0.06%) compartments by MRI, adjusted for age, gender, BMI and K-L grade. A higher baseline WOMAC pain score was also associated with increased incidence of ROA (OR 1.07, 95% CI 1.01–1.13) (top half of Table 3). In ROA, a greater baseline WOMAC pain score was associated with increased rate of cartilage volume loss in medial (regression coefficient 0.04%, 95% CI 0.02–0.07%) and lateral (0.05%, 0.03–0.07%) compartments by MRI. A higher baseline WOMAC pain score was also associated with increased progression of ROA (OR 1.07, 95% CI 1.03–1.10) (bottom half of Table 3).

**Table 3:** Associations of baseline Western Ontario and McMaster Universities Osteoarthritis Index knee pain score with annual percentage cartilage volume loss and incidence and progression of radiographic knee osteoarthritis over 4 years

	Univariable analysis		Multivariable analysis^a^	
Non-ROA				
	Regression coefficient (95% CI)	*p* Value	Regression coefficient (95% CI)	*p* Value
Annual percentage cartilage volume loss in medial compartment	0.04 (0.01, 0.06)	0.001	0.04 (0.02, 0.06)	0.001
Annual percentage cartilage volume loss in lateral compartment	0.04 (0.02, 0.06)	< 0.001	0.04 (0.02, 0.06)	< 0.001
	OR (95% CI)	*p* Value	OR (95% CI)	*p* Value
Incidence of radiographic knee osteoarthritis	1.10 (1.05, 1.16)	< 0.001	1.07 (1.01, 1.13)	0.02
ROA				
	Regression coefficient (95% CI)	*p* Value	Regression coefficient (95% CI)	*p* Value
Annual percentage cartilage volume loss in medial compartment	0.06 (0.03, 0.08)	< 0.001	0.04 (0.02, 0.07)	0.001
Annual percentage cartilage volume loss in lateral compartment	0.06 (0.04, 0.08)	< 0.001	0.05 (0.03, 0.07)	< 0.001
	OR (95% CI)	*p* Value	OR (95% CI)	*p* Value
Progression of radiographic knee osteoarthritis	1.08 (1.05, 1.12)	< 0.001	1.07 (1.03, 1.10)	< 0.001

*ROA* Radiographic osteoarthritis

^a^Adjusted for age, gender, body mass index and Kellgren-Lawrence grade

### Associations between knee pain patterns over 1 year and structural progression over 4 years

In non-ROA, the annual rate of cartilage volume loss in the medial compartment was 0.63% (SE 0.03%) in participants with no knee pain, 0.81% (0.08%) in those with fluctuating knee pain, and 0.93% (0.12%) in those with persistent knee pain (*p* for trend = 0.003), adjusted for age, gender, BMI, and K-L grade (Table 4). The rate was greater in participants with fluctuating (*p* = 0.04) and persistent (*p* = 0.02) knee pain than in those without knee pain. Similar results were found in the lateral compartment. Although persistent knee pain was not significantly associated with the incidence of ROA (OR 1.57, 95% CI 0.85–2.90), fluctuating knee pain was associated with increased incidence of ROA (OR 1.62, 95% CI 1.04–2.54; *p* for trend = 0.03) (Table 4). The attributable risk of fluctuating knee pain for incident ROA was 35% (4–55%), with an NNH of 19.5.

**Table 4:** Associations of knee pain patterns over 1 year with annual percentage cartilage volume loss and incidence of radiographic knee osteoarthritis over 4 years in participants without radiographic knee osteoarthritis at baseline

	Univariable analysis		Multivariable analysis^a^	
	Estimated marginal mean (SE)	*p* Value	Estimated marginal mean (SE)	*p* Value
Annual percentage cartilage volume loss in medial compartment				
No knee pain at both baseline and 1 year	0.63 (0.03)		0.63 (0.03)^b,c^	
Fluctuating knee pain (pain at either time point)	0.80 (0.08)	0.01^d^	0.81 (0.08)^b^	0.01^d^
Persistent knee pain (pain at both time points)	0.94 (0.12)		0.93 (0.12)^c^	
Trend		0.004		0.003
Annual percentage cartilage volume loss in lateral compartment				
No knee pain at both baseline and 1 year	0.68 (0.03)		0.68 (0.03)^e,f^	
Fluctuating knee pain (pain at either time point)	0.88 (0.08)	0.008^d^	0.89 (0.08)^e^	0.005^d^
Persistent knee pain (pain at both time points)	0.92 (0.11)		0.93 (0.11)^f^	
Trend		0.003		0.002
	OR (95% CI)	*p* Value	OR (95% CI)	*p* Value
Incidence of radiographic knee osteoarthritis				
No knee pain at both baseline and 1 year	1.00		1.00	
Fluctuating knee pain (pain at either time point)	1.96 (1.29, 2.99)	0.002	1.62 (1.04, 2.54)	0.03
Persistent knee pain (pain at both time points)	2.07 (1.16, 3.72)	0.01	1.57 (0.85, 2.90)	0.15
Trend		< 0.001		0.03

^a^Adjusted for age, gender, body mass index and Kellgren-Lawrence grade

^b^*p* = 0.04 for between-group difference

^c^*p* = 0.02 for between-group difference

^d^For difference in annual percentage cartilage volume loss in medial/lateral compartment among the three knee pain pattern groups

^e^*p* = 0.01 for between-group difference

^f^*p* = 0.03 for between-group difference

In ROA, the annual rate of cartilage volume loss in the medial compartment was 1.26% (SE 0.06%) in participants with no knee pain, 1.47% (0.11%) in those with fluctuating knee pain, and 1.60% (0.12%) in those with persistent knee pain (*p* for trend = 0.01), adjusted for age, gender, BMI and K-L grade (Table 5). The rate was greater in participants with persistent (*p* = 0.02) knee pain than in those without knee pain. Similar results were shown in the lateral compartment. Although fluctuating knee pain was not significantly associated with the progression of ROA (OR 1.34, 95% CI 0.94–1.89), persistent knee pain was associated with increased progression of ROA (OR 1.82, 95% CI 1.28–2.60; *p* for trend = 0.001) (Table 5). The attributable risk of persistent knee pain for progressive ROA was 37% (18–50%), corresponding to the NNH of 9.6.

**Table 5:** Associations of knee pain patterns over 1 year with annual percentage cartilage volume loss and progression of radiographic knee osteoarthritis over 4 years in participants with radiographic knee osteoarthritis at baseline

	Univariable analysis		Multivariable analysis^a^	
	Estimated marginal mean (SE)	*p* Value	Estimated marginal mean (SE)	*p* Value
Annual percentage cartilage volume loss in medial compartment				
No knee pain at both baseline and 1 year	1.22 (0.06)		1.26 (0.06)^b^	
Fluctuating knee pain (pain at either time point)	1.52 (0.11)	0.001^c^	1.47 (0.11)	0.03^c^
Persistent knee pain (pain at both time points)	1.68 (0.12)		1.60 (0.12)^b^	
Trend		< 0.001		0.01
Annual percentage cartilage volume loss in lateral compartment				
No knee pain at both baseline and 1 year	1.06 (0.05)		1.08 (0.05)^d,e^	
Fluctuating knee pain (pain at either time point)	1.41 (0.08)	< 0.001^c^	1.38 (0.08)^d^	< 0.001^c^
Persistent knee pain (pain at both time points)	1.46 (0.09)		1.39 (0.09)^e^	
Trend		< 0.001		< 0.001
	Odds ratio (95% CI)	*p* Value	Odds ratio (95% CI)	*p* Value
Progression of radiographic knee osteoarthritis				
No knee pain at both baseline and 1 year	1.00		1.00	
Fluctuating knee pain (pain at either time point)	1.46 (1.04, 2.06)	0.03	1.34 (0.94, 1.89)	0.10
Persistent knee pain (pain at both time points)	2.13 (1.53, 2.97)	< 0.001	1.82 (1.28, 2.60)	0.001
Trend		< 0.001		0.001

*Adjusted for age, gender, body mass index and Kellgren-Lawrence grade

^b^*p* = 0.02 for between-group difference

^c^ For difference in annual percentage cartilage volume loss in medial/lateral compartment among the three knee pain pattern groups

^d^*p* = 0.001 for between-group difference

^e^*p* = 0.002 for between-group difference

Similar results were observed for the association between knee pain patterns over 2 and 3 years and structural progression over 4 years (Additional file 1: Tables S1 and S2).

## Discussion

In this large prospective cohort study, greater baseline knee pain, as well as fluctuating and persistent knee pain over 1 year, predicted structural progression over 4 years in participants with and without knee ROA, as evidenced by accelerated cartilage volume loss and increased incidence and progression of ROA. Among non-ROA participants with fluctuating knee pain over 1 year, 35% of the incident ROA risk over 4 years could be attributed to fluctuating knee pain, corresponding to an NNH of 19.5. In participants with ROA with persistent knee pain over 1 year, 37% of the progressive ROA risk over 4 years could be attributed to persistent knee pain, corresponding to an NNH of 9.6. These data suggest that knee pain is an important predictive factor for the deterioration of knee structural outcomes and highlight the significant adverse impact of persistent knee pain on knee structures. The findings suggest that treating patients with knee pain both early in the disease course and over time is important for preserving knee structure and is likely to have a significant impact on reducing disease burden.

There have been conflicting data on the association between knee pain and structural progression of knee OA [1, 5, 6, 10, 12–19]. This may have resulted from limited sample sizes and durations of follow-up in both MRI and radiological studies. Cohort studies with larger sample sizes and/or longer durations of follow-up have shown significant associations of knee pain with MRI [5, 14] and radiographic [16, 18, 19] outcomes. One cohort study analysing baseline and 12-month follow-up data in a large subsample of 718 participants with K-L grades 2–4 from the OAI showed knees with frequent pain had greater rates of cartilage thickness loss in the central subregion of the medial femoral condyle than knees without pain [5]. Our study of a large knee ROA cohort with 4 years of follow-up showed that higher levels of baseline knee pain predicted increased structural progression over 4 years assessed by both MRI (cartilage volume loss) and x-ray (progression of ROA). Our study extended the previous OAI study [5] by examining baseline knee pain as a continuous variable, thus indicating a dose-response relationship; by investigating knee pain pattern over time and its association with structural outcomes, showing associations for cartilage volume loss of both medial and lateral tibiofemoral compartments and consistent results for MRI and radiographic outcomes; and by examining participants with and without knee ROA simultaneously. The effect sizes for some of the associations appeared small, particularly for those between baseline WOMAC knee pain score and annual percentage cartilage volume loss (Table 3). However, it is important to put them in context. For example, in those with no ROA, even small changes in the annual rate of cartilage volume loss for small changes in knee pain will have significant impacts over many years. Only three studies have examined the relationship between knee pain and structural changes in non-OA populations [11, 16, 19]. Although one study found no association between baseline knee pain and tibial cartilage volume loss over 2.5 years [11], the other reported baseline knee pain being associated with incident ROA defined by K-L ≥ 1 but not K-L ≥ 2 over 5.1 years [16] and incident accelerated knee OA over 4 years [19]. We defined incident ROA using the more stringent K-L grade ≥ 2 at follow-up. Our large 4-year follow-up study of participants with no ROA demonstrated that baseline knee pain predicted cartilage volume loss and incidence of ROA, suggesting a predictive role of knee pain in adverse structural outcomes, even in people without knee ROA.

Given the fluctuating nature of knee pain, we examined the association between knee pain patterns over 1 year and structural progression over 4 years. We found that fluctuating and persistent knee pain over 1 year predicted increased cartilage volume loss as well as incidence and progression of ROA over 4 years in participants with and without ROA, with positive linear relationships observed between the frequency of knee pain over 1 year and structural progression over 4 years. Previous studies reported that worsening of knee pain was associated with increased cartilage volume loss simultaneously (i.e., over the same time period) [1, 12, 13]. The adverse effect of ongoing (fluctuating or persistent) knee pain on knee structure has not previously been examined. We found that fluctuating and persistent knee pain over 1 year contributed substantially to the incidence and progression of ROA over 4 years. For every 20 non-ROA participants with fluctuating knee pain over 1 year, 1 developed incident ROA in 4 years. For every ten ROA participants with persistent knee pain over 1 year, one had progressive ROA in 4 years. These findings support the importance of controlling knee pain over time and that targeting people with fluctuating or persistent knee pain for early intervention will be important for preserving knee structure and delaying structural progression.

The mechanism of knee pain and structural change is likely to be multifactorial. Optimal knee function requires a complex interplay of structural and biomechanical factors, including supporting musculature. It has been shown that increased size and decreased fat content of the vastus medialis predict reduced cartilage volume loss, and the most significant predictor of increased muscle size is an improvement in knee pain [27–29]. Nonetheless, the causes of knee pain are complex and heterogeneous and include structural factors [30], inflammatory hyperalgesia [31], central mechanisms incorporating brain areas processing fear, emotions and in aversive conditioning [32], and genetic predispositions toward peripheral pain sensitisation [33]. There is evidence that inflammation [8, 9] and other structural abnormalities, such as bone marrow lesions and effusion-synovitis [34–36], are associated with greater knee pain and structural progression. Greater K-L grade is associated with higher levels of knee pain [30] and accelerated cartilage loss [37]. However, it is less likely that our findings are explained by disease severity, because all the analyses were adjusted for baseline K-L grade, and the outcome of annual percentage loss in cartilage volume took into account the baseline amount of cartilage volume. Understanding and appropriately targeting the factors related to knee pain in an individual may be particularly important for modifying disease trajectory, such as targeting quadriceps strengthening, bone marrow lesions, synovitis, central sensitisation, or weight loss.

This study has limitations. The selection of target knee for each participant was based on radiographic severity. It is more likely that this target knee is the one contributing to pain, but it is also possible that knee pain originates from the other knee and that there has been switching. This would have underestimated the magnitude of observed associations. There is also evidence that pain in one knee has adverse effects on the other knee through compensatory gait mechanisms that shift the load distribution from the affected limb to the healthy contralateral limb during weight-bearing activities [38, 39]. We did not adjust our results for medications that may influence pain over time, because the information was obtained by questionnaire, and the answers were limited. However, any symptoms experienced by the participants, including fluctuating symptoms, would have been present despite any therapies, so the notion of pain predicting structural progression remains valid. Furthermore, knee pain was analysed prior to the assessment of structural outcomes, thus it is unlikely that these results can be explained simply by structural changes causing pain. Although there was potential selection bias in the study, in which the non-completers had higher K-L grade and worse knee pain than the completers, this would not affect the interpretation of our results. We did not examine other structural abnormalities associated with pain, such as bone marrow lesions and effusion-synovitis [40–42], but this does not affect the interpretation of our findings, because such structural alterations are likely to be on the causal pathway from knee pain to structural outcomes [43–45]. There was an issue of multiple testing in our analyses. For each of the incidence cohort and the progression cohort, we examined two knee pain variables and three outcome variables, and thus we performed six tests. If we performed the Bonferroni correction, the significance level should be 0.05/6 = 0.008. Most of our results remained significant after applying the Bonferroni correction. However, it is important to consider that we did not perform completely independent, unrelated analyses, because both cartilage volume loss and radiographic changes are on the same disease pathway. We found consistent results for incidence and progression of ROA, as well as for MRI and radiographic outcomes, after adjustment for potential confounders, suggesting a true association rather than a chance association. There is also the possibility of residual confounding that knee pain could be a biomarker of other unmeasured factors which are associated with structural progression.

The present study has several strengths. The OAI offered a unique opportunity to study the disease profile of a large number of participants and explore the impact of knee pain on structural progression. Until now, the assessment of cartilage volume change by quantitative MRI has mostly been done using manual or semi-automated technologies, which have the intrinsic limitation of variability in results with respect to human intervention. This imposed limitations on a complete analysis of the OAI cohort. The validation of fully automated technology to assess cartilage volume and its change over time [26] has greatly improved the capacity and reliability of the analysis of the OAI MRI dataset. We examined whether knee pain predicted structural endpoints in people with and without ROA. The OAI intentionally recruited participants at risk of knee OA. This enriched the population for the outcome of interest and thus increased the power of the study to detect significant associations between knee pain and structural outcomes. Knee pain was assessed at baseline and 1 year later using a valid questionnaire, from which knee pain patterns were investigated with a positive linear relationship observed between the frequency of knee pain over 1 year and structural progression over 4 years. This is important because knee pain can fluctuate with time, and thus an isolated baseline assessment may have limited effect on predicting structural progression many years later.

## Conclusions

Greater baseline knee pain, as well as fluctuating and persistent knee pain over 1 year, predicted increased cartilage volume loss and incidence of ROA in people with no ROA and increased cartilage volume loss and progression of ROA in those with ROA over 4 years. With its large cohort, this study provides evidence that knee pain is an important predictor of structural disease progression in population-based individuals with and without knee OA. This study suggests that controlling knee pain early in the disease course as well as over time by targeting the underlying mechanisms may be important for preserving knee structure and reducing the burden of knee OA. Further studies are needed to determine if this is the case.

## Additional file

Additional file 1: **Table S1.** Associations of knee pain patterns over 2 and 3 years with structural outcomes over 4 years in participants without radiographic knee osteoarthritis at baseline. **Table S2.** Associations of knee pain patterns over 2 and 3 years with structural outcomes over 4 years in participants with radiographic knee osteoarthritis at baseline. (DOCX 21 kb)

## Abbreviations

BMI: Body mass index
K-L: Kellgren-Lawrence
MRI: Magnetic resonance imaging
NNH: Number needed to harm
OA: Osteoarthritis
OAI: Osteoarthritis Initiative
ROA: Radiographic osteoarthritis
VAS: Visual analogue scale
WOMAC: Western Ontario and McMaster Universities Osteoarthritis Index
