# Supplementary material for: Knee pain as a predictor of structural progression over 4 years: data from the Osteoarthritis Initiative, a prospective cohort study
Source: Arthritis Res Ther. 2018 Nov 6;20:250. doi: 10.1186/s13075-018-1751-4 (PMC6235215; doi:10.1186/s13075-018-1751-4)
Supplement: Supplementary file 1 — Table S1. Associations of knee pain patterns over 2 and 3 years with structural outcomes over 4 years in participants without radiographic knee osteoarthritis at baseline. Table S2. Associations of knee pain patterns over 2 and 3 years with structural outcomes over 4 years in participants with radiographic knee osteoarthritis at baseline. (DOCX 21 kb) [file 13075_2018_1751_MOESM1_ESM.docx]

**Table S1: Associations of knee pain patterns over 2 and 3 years with structural outcomes over 4 years in participants without radiographic knee osteoarthritis at baseline**

|  | **Knee pain patterns over 2 years** | | **Knee pain patterns over 3 years** | |
| --- | --- | --- | --- | --- |
|  | **Estimated marginal mean (SE)** | **P** | **Estimated marginal mean (SE)** | **P** |
| **Annual % cartilage volume loss in medial compartment** | |  |  |  |
| No knee pain at both time points | 0.61 (0.03)^1^ |  | 0.61 (0.03)^5,6^ |  |
| Fluctuating knee pain (pain at either time point) | 0.75 (0.07)^2^ | <0.001⁋ | 0.75 (0.06)^5,7^ | <0.001⁋ |
| Persistent knee pain (pain at both time points) | 1.36 (0.17)^1,2^ |  | 1.47 (0.19)^6,7^ |  |
| Trend |  | <0.001 |  | <0.001 |
| **Annual % cartilage volume loss in lateral compartment** | |  |  |  |
| No knee pain at both time points | 0.67 (0.03)^3^ |  | 0.66 (0.03)^8^ |  |
| Fluctuating knee pain (pain at either time point) | 0.79 (0.06)^4^ | <0.001⁋ | 0.78 (0.06)^9^ | <0.001⁋ |
| Persistent knee pain (pain at both time points) | 1.37 (0.16)^3,4^ |  | 1.48 (0.17)^8,9^ |  |
| Trend |  | <0.001 |  | <0.001 |
|  | **Odds ratio (95% CI)** | **P** | **Odds ratio (95% CI)** | **P** |
| **Incidence of radiographic knee osteoarthritis** | |  |  |  |
| No knee pain at both time points | 1.00 |  | 1.00 |  |
| Fluctuating knee pain (pain at either time point) | 1.80 (1.22, 2.67) | 0.003 | 1.95 (1.34, 2.84) | <0.001 |
| Persistent knee pain (pain at both time points) | 2.02 (0.91, 4.47) | 0.08 | 1.72 (0.71, 4.16) | 0.23 |
| Trend |  | 0.002 |  | 0.001 |

All analyses adjusted for age, gender, body mass index, and Kellgren-Lawrence grade

⁋For difference in annual % cartilage volume loss in medial/lateral compartment among the three knee pain pattern groups

^1^p<0.001, ^2^p=0.001, ^3^p<0.001, ^4^p=0.001, ^5^p=0.04, ^6^p<0.001, ^7^p<0.001, ^8^p<0.001, ^9^p<0.001 for between-group difference

**Table S2: Associations of knee pain patterns over 2 and 3 years with structural outcomes over 4 years in participants with radiographic knee osteoarthritis at baseline**

|  | **Knee pain patterns over 2 years** | | **Knee pain patterns over 3 years** | |
| --- | --- | --- | --- | --- |
|  | **Estimated marginal mean (SE)** | **P** | **Estimated marginal mean (SE)** | **P** |
| **Annual % cartilage volume loss in medial compartment** | |  |  |  |
| No knee pain at both time points | 1.22 (0.07)^1^ |  | 1.18 (0.07)^4^ |  |
| Fluctuating knee pain (pain at either time point) | 1.60 (0.09)^1^ | 0.004⁋ | 1.62 (0.09)^4^ | <0.001⁋ |
| Persistent knee pain (pain at both time points) | 1.45 (0.14) |  | 1.40 (0.16) |  |
| Trend |  | 0.01 |  | 0.006 |
| **Annual % cartilage volume loss in lateral compartment** | |  |  |  |
| No knee pain at both time points | 1.07 (0.05)^2,3^ |  | 1.06 (0.05)^5,6^ |  |
| Fluctuating knee pain (pain at either time point) | 1.38 (0.07)^2^ | 0.001⁋ | 1.35 (0.06)^5^ | 0.001⁋ |
| Persistent knee pain (pain at both time points) | 1.32 (0.10)^3^ |  | 1.33 (0.11)^6^ |  |
| Trend |  | 0.001 |  | 0.001 |
|  | **Odds ratio (95% CI)** | **P** | **Odds ratio (95% CI)** | **P** |
| **progression of radiographic knee osteoarthritis** | |  |  |  |
| No knee pain at both time points | 1.00 |  | 1.00 |  |
| Fluctuating knee pain (pain at either time point) | 1.58 (1.16, 2.16) | 0.004 | 1.83 (1.35, 2.48) | <0.001 |
| Persistent knee pain (pain at both time points) | 2.03 (1.36, 3.04) | 0.001 | 2.31 (1.48, 3.62) | <0.001 |
| Trend |  | <0.001 |  | <0.001 |

All analyses adjusted for age, gender, body mass index, and Kellgren-Lawrence grade

⁋For difference in annual % cartilage volume loss in medial/lateral compartment among the three knee pain pattern groups

^1^p=0.001, ^2^p<0.001, ^3^p=0.03, ^4^p<0.001, ^5^p<0.001, ^6^p=0.03 for between-group difference
